# Clinical applicability of miR517a detection in liquid biopsies of ETMR patients

**DOI:** 10.1007/s00401-023-02567-z

**Published:** 2023-04-05

**Authors:** Sibylle Madlener, Julia Furtner, Natalia Stepien, Daniel Senfter, Lisa Mayr, Maximilian Zeyda, Leon Gramss, Barbara Aistleitner, Sabine Spiegl-Kreinecker, Elisa Rivelles, Christian Dorfer, Karl Rössler, Thomas Czech, Amedeo A. Azizi, Andreas Peyrl, Daniela Lötsch-Gojo, Leonhard Müllauer, Christine Haberler, Irene Slavc, Johannes Gojo

**Affiliations:** 1 Department of Pediatrics and Adolescent Medicine, Comprehensive Center for Pediatrics and Comprehensive Cancer Center, Medical University of Vienna, Vienna, Austria; 2 Department of Biomedical Imaging and Image-Guided Therapy, Medical University of Vienna, Vienna, Austria; 3 Research Center of Image Analysis and Artificial Intelligence (MIAAI), Danube Private University, Krems-Stein, Austria; 4 Department of Pediatrics and Adolescent Medicine, Johannes Kepler University Linz, Linz, Austria; 5 Department of Neurosurgery, Kepler University Hospital GmbH, Johannes Kepler University, Linz, Austria; 6 Department of Laboratory Medicine, Medical University of Vienna, Vienna, Austria; 7 Department of Neurosurgery, Medical University of Vienna, Vienna, Austria; 8 Department of Pathology, Medical University of Vienna, Vienna, Austria; 9 Division of Neuropathology and Neurochemistry, Department of Neurology, Medical University of Vienna, Vienna, Austria

Embryonal tumor with multilayered rosettes (ETMR) is a distinct central nervous system tumor entity which is characterized by dysregulation of oncogenic micro RNAs (miRNA) [2, 7]. In the majority of cases (approx. 90%), tumors are characterized by amplification of the *C19MC* locus accompanied by a fusion of this locus to the *TTYH1* gene [7]. Approximately 10% are *C19MC*-negative and either harbor amplification of the miRNA cluster on chromosome 17 (MIR17HG) or bi-allelic mutations in *DICER1*, a central component of the miRNA processing machinery [6]. Tumors are usually detected by magnetic resonance imaging (MRI) and are mostly already large tumors having metastasized at diagnosis in approximately one-quarter of cases [3–5, 10]. The definitive diagnosis is based on histopathological (morphology, LIN28A positivity) and molecular analyses (DNA methylation, *C19MC* amplification) of tumor tissue [2]. ETMRs predominantly affect very young children and show a dismal clinical outcome, as about half of the patients relapse within the first 6 months despite intensive therapy [5]. Only one-quarter of patients survives longer than 2 years [3, 5]. Based on these facts, a more accurate and faster detection of tumor response or tumor relapse would significantly improve the management of these severely affected young patients. Moreover, MRIs can frequently only be performed in general anesthesia in this patient population resulting in further burden for patients.

Liquid biopsy has emerged as a highly promising tool to enable minimal invasive detection of molecular parameters to facilitate diagnosis and patient monitoring [8]. To develop liquid biopsy tools for ETMR, we performed a comprehensive analysis of cell-free DNA as well as miRNA in cerebrospinal fluid and blood of ETMR patients (Fig. 1a). Our patient cohort comprised tumor tissue of ten *C19MC*-amplified cases and one *DICER1* mutated ETMR case which we in part previously published [9]. In addition, we included matched cerebrospinal fluid (six cases) and blood (three plasma and two serum) liquid biopsy samples as well as three dried blood spots from the Austrian newborn screening program. First, we screened tumor tissues (*n* = 10) for upregulated miRNAs applying the nCounter miRNA expression assay (NanoString), covering more than 800 known tumor-associated miRNAs and detected a specific miRNA pattern in our cohort. *C19MC*-amplified ETMR tissues expressed a unique pattern of more than 50 miRNAs, showing a distinct miRNA expression pattern as compared to other pediatric brain tumors (Fig. 1b, c). Of the *C19MC*-associated miRNAs, miR517a was the most significantly overexpressed miRNA (Fig. 1d). To develop a robust and easily applicable miRNA detection method, we established a qRT-PCR method to detect miR517a and validated its expression across 11 ETMR tumor tissues as well as controls (Fig. 1e). MiR517a expression in *C19MC*-amplified ETMR showed a fold change at a minimum of 200 and a maximum of 16 000, whereas the *DICER1* mutated case (case#3) as well as tumor and epilepsy brain controls had significantly lower expression levels ranging from 0.04 up to 100 (Fig. 1d, e). Next, we analyzed blood samples of five different cases, each at time points with radiological evidence of tumor. MiR517a was significantly higher in blood of ETMR patients as compared to samples of non-tumor bearing controls and of medulloblastoma patients (MB) (Fig. 1f). Interestingly, detected expression levels were almost of the same magnitude as in tumor tissues (120–16.000 fold change). Subsequently, we screened different liquid biopsy samples (CSF, blood) using our newly developed qRT-PCR for miR517a as well as digital-droplet PCR (ddPCR) for C19MC amplification detection (Fig. 1g). We detected upregulated CNV rates in cfDNA obtained from CSF, whereas elevated miR517a levels were only present in blood samples. Based on these results, we determined receiver operating characteristic (ROC) curves showing an area under the curve of (AUC) 0.835 for CNV in CSF (Fig. 1 h), 0.627 for miR517a in CSF (Fig. 1i), and 1.0 for miR517a detection in blood samples (Fig. 1j). Accordingly, detection of elevated miR517a levels in blood showed the highest sensitivity (100%) and specificity (100%) (Supplementary Tab. 2). Importantly, we observed no difference between plasma and serum samples (Supplementary Tab. 2). As already mentioned, ETMRs predominantly arise in very young children and have even been described in neonates [1, 5]. This suggests that tumors or precursor lesions may be already present at birth. Consequently, we sought to investigate whether we could apply miR517a or C19MC amplification detection in dry blot spots obtained within neonatal screening. In three cases who were later diagnosed with ETMR (age 27–38 months), we could analyze dry blood spots from the Austrian newborn screening program. However, we could neither detect expression of miR517a for RNA (data not shown) nor C19M﻿C amplification (Supplementary Fig. 1). Consequently, our limited analysis of dried blood spots at newborn screening did not prove feasibility for screening of children at risk for developing ETMR.

**Fig. 1:**
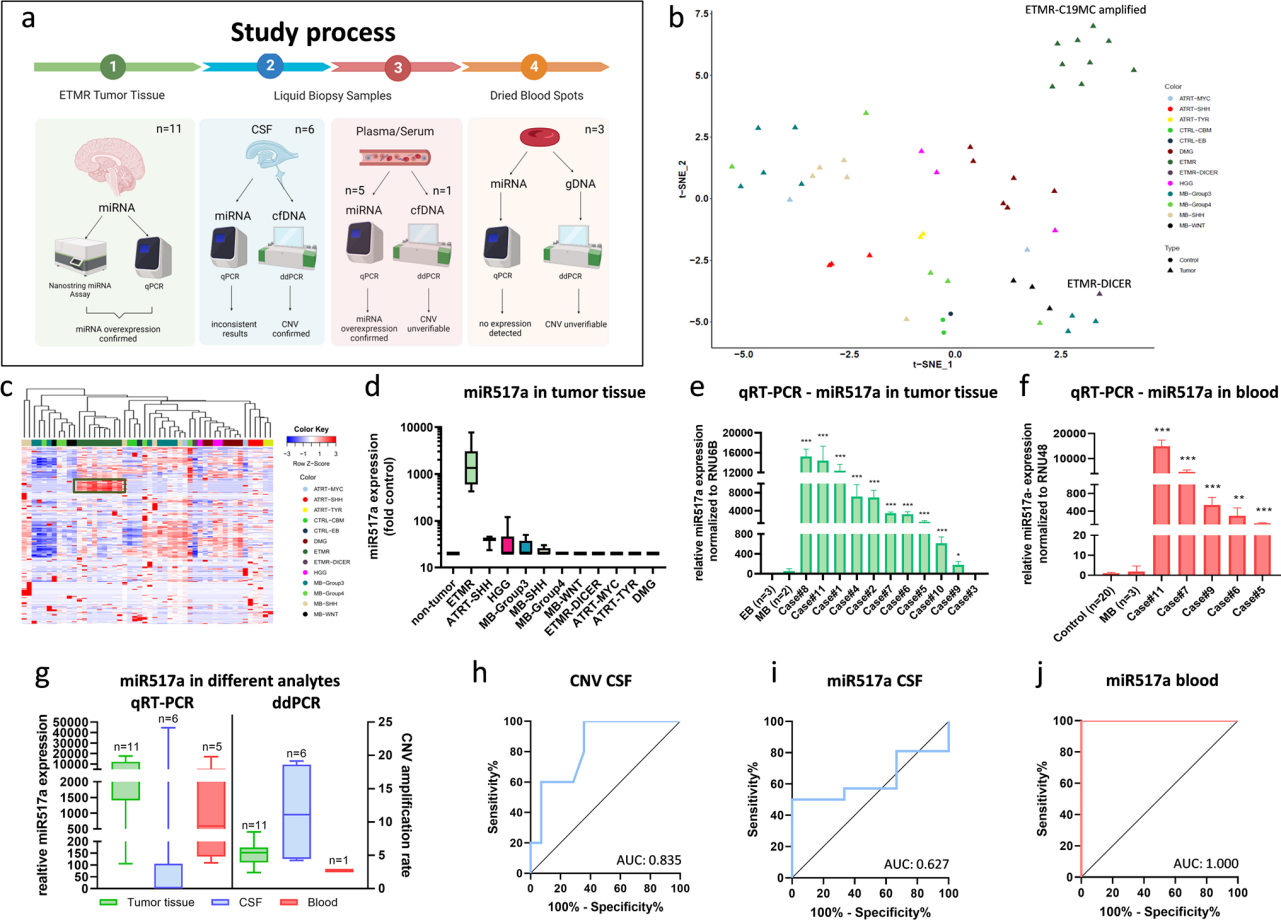
Blood-derived miR517a is a specific biomarker for ETMR. **a** Graphical abstract of the study (created with BioRender). **b** tSNE clustering of NanoString miRNA data. **c** Heatmap of NanoString analysis of more than 800 miRNAs expressed in different brain tumors. **d** MiR517a expression in pediatric brain tumor tissues (NanoString data). **e** miR517a expression in ETMR tissues and controls measured by qRT-PCR. **f** miR517a expression in blood samples of ETMR patients. **g** CNV and miR517a expression detected in different analytes. **h** Receiver operating characteristic (ROC) of miR517a CNV in CSF samples. **i** ROC curve of miR517a expression in CSF samples. **j** ROC curve of miR517a in blood samples. NanoString analysis was performed with nSolver software 3.0 from NanoString. t SNE was calculated with r Studio. All qPCR experiments were performed in triplicates. Asterisks indicate significance (students *t* test; *P < 0.05, **P < 0.01, ***P < 0.001 analyzed with GraphPad Prism), and error bars indicate that mean ± S.D. ROC curves were analyzed in IBM SPSS statistics 27 and mapped with GraphPad Prism

Based on the promising results for detection in blood, we tested whether serial miR517a measurements harbored potential not only for diagnostic purposes, but also for longitudinal tumor monitoring. Strikingly, the levels of miR517a significantly correlated with tumor volume (Pearson correlation coefficient 0.995, *p* > 0.001). We further correlated miR517a levels to the individual patient histories and could show that levels decreased after tumor resection (Fig. 2a, Supplementary Fig. 2b) and in long-term survivors (Fig. 2b, Supplementary Fig. 2a). Moreover, we observed an increase in miR517a levels upon tumor progression (Fig. 2c). In addition, we analyzed C19MC amplification in CSF at selected time points only showing an increase at tumor recurrence in one case (Fig. 2a, c; Supplementary Fig. 2a). Figure 2d summarizes the time points of liquid biopsy in relation to the clinical course of the individual patients. Patient treatments are outlined in Supplementary Table 3.

**Fig. 2:**
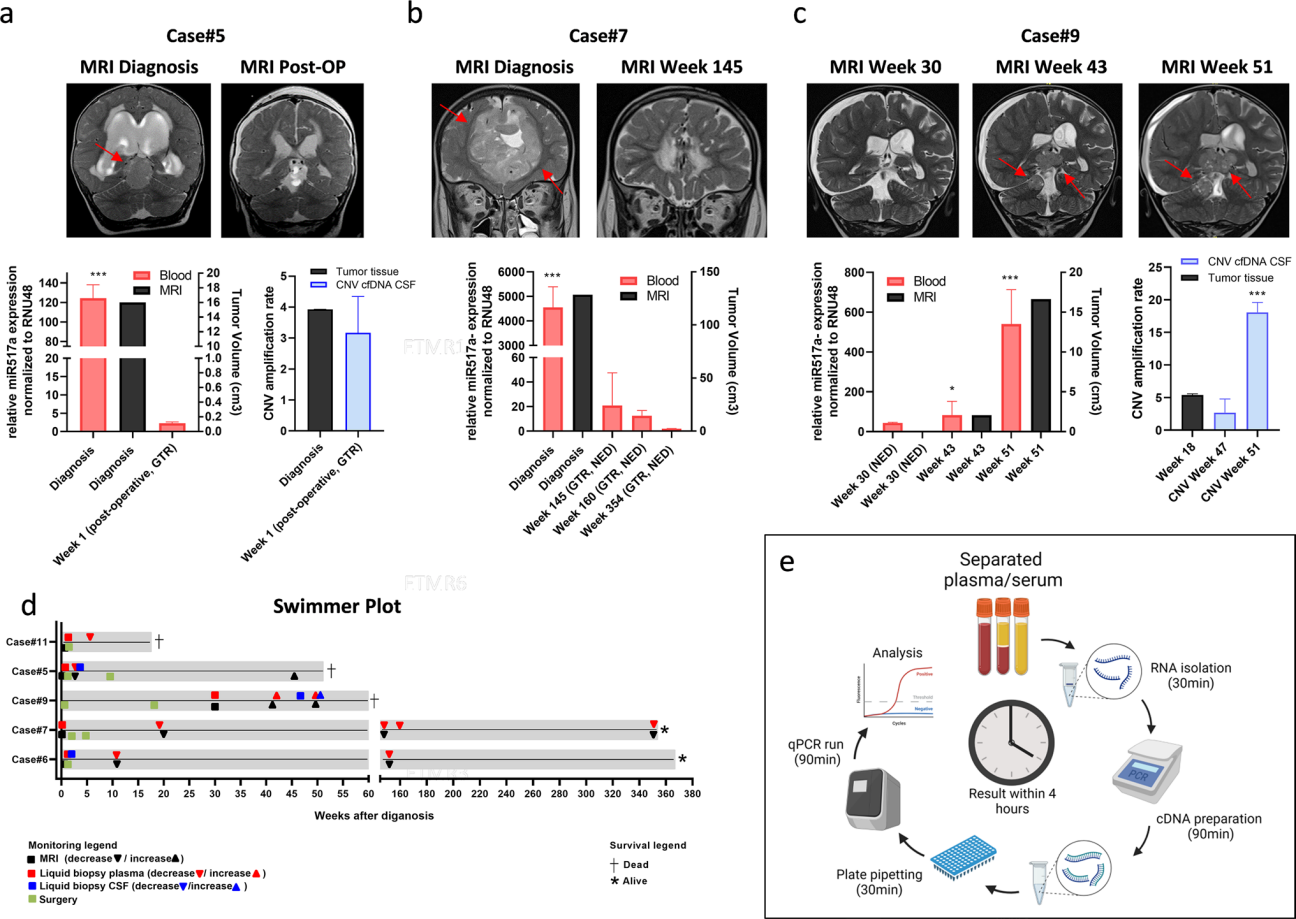
Liquid biopsy detection of miRNA enables therapy monitoring in ETMR patients. **a**–**c** Different ETMR cases and matched MRI images (FLAIR, upper panel) as well as longitudinal monitoring of miR517a expression in serum for case#5 and plasma for case#7 and case#9 correlated to tumor volumes (lower panel). CNV of miR517a detected in tumor DNA and cfDNA from CSF for case#5 and case#9. **d** Swimmer plot of 5 ETMR patients, including all dates of surgery, liquid biopsies, and MRIs. **e** Illustration of experimental workflow (created with BioRender). All qPCR experiments were performed in triplicates. Asterisks indicate significance (Student’s *t* test; *P < 0.05, **P < 0.01, ***P < 0.001), and error bars indicate mean ± S.D. GTR, gross total resection; NED, no evidence of disease

In summary, we developed a reliable and robust method for rapid detection of tumor-derived miRNA in ETMR patients (Fig. 2d). We want to highlight that the test results are generated with minimal costs and equipment as well as within a turnaround time of only 4 h. This short period from blood collection to diagnosis combined with the robustness emphasizes the high potential of this newly developed miRNA biomarker. This is in agreement with the well-described stability of miRNA in body fluids and potentially facilitated by inclusion of miR517a in small vesicles or protein complexes [8, 11]. With respect to the limited patient number, a validation of the presented biomarker in a larger patient cohort is suggested. Taking into account the young age of ETMR patients and potentially rapid changes in the disease course, our widely applicable method could substantially impact the possibilities of diagnosis and therapy monitoring in this highly aggressive tumor type.

## Supplementary Information

Below is the link to the electronic supplementary material.

Supplementary file1 (PDF 396 kb)

Supplementary file2 (PDF 120 kb)

## Data Availability

The data presented in this study is contained within the presented figures and supplementary figures. Any additional data is available on request from the corresponding author, due to privacy restrictions.
