# Supplementary material for: Clinical applicability of miR517a detection in liquid biopsies of ETMR patients
Source: Acta Neuropathol. 2023 Apr 5;145(6):843–6. doi: 10.1007/s00401-023-02567-z (PMC10175469; doi:10.1007/s00401-023-02567-z)
Supplement: Supplementary file 1 — Supplementary file1 (PDF 396 kb) [file 401_2023_2567_MOESM1_ESM.pdf]

Clinical applicability of miR517a detection in liquid biopsies of ETMR patients

Madlener et al.

Online supplementary material

Supplementary Figures 1-3

Supplementary Tables 1-3

Supplementary Figure 1

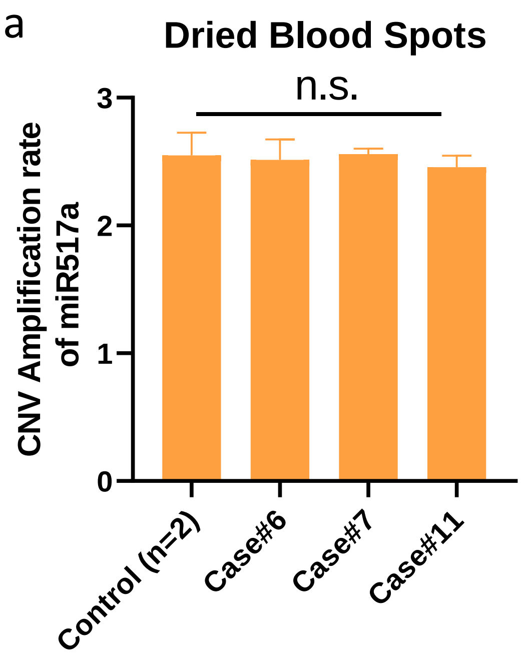

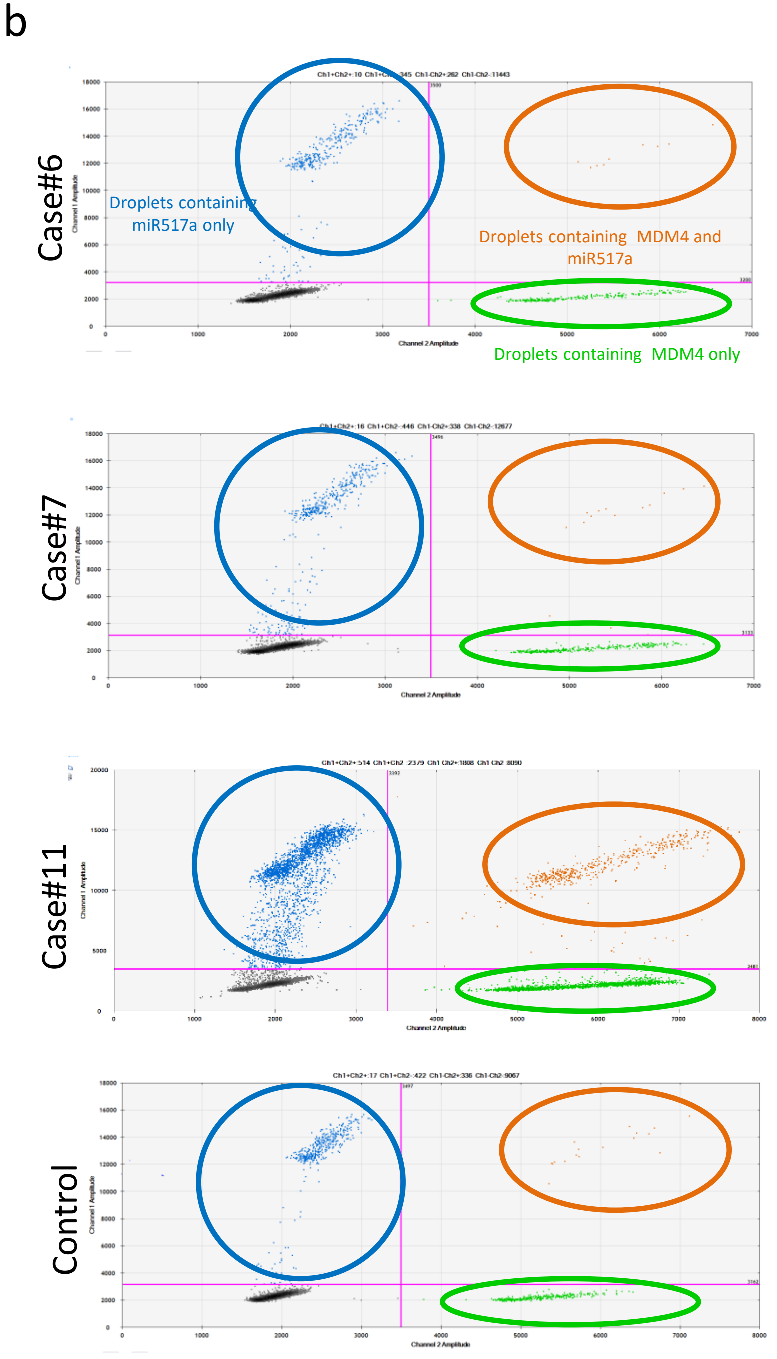

Supplementary Fig. 1: a) Copy number variation of miR517a in dried blood spots. The amplification cut off was set to 2.5 b) Scatter plot of ddPCR, blue is representing positive droplets for miR517a, orange are double positive droplets including referenece gene (MDM4) and miR517a, green are positive droplets for the reference gene and grey droplets were negative (no DNA detected). The amplification rate was calculated as followed:

$$\text{Amplification rate} = \left(\frac{\text{Copies gene of interest}}{\text{Copies of reference gene}}\right) \text{x2}$$

Supplementary Figure 2

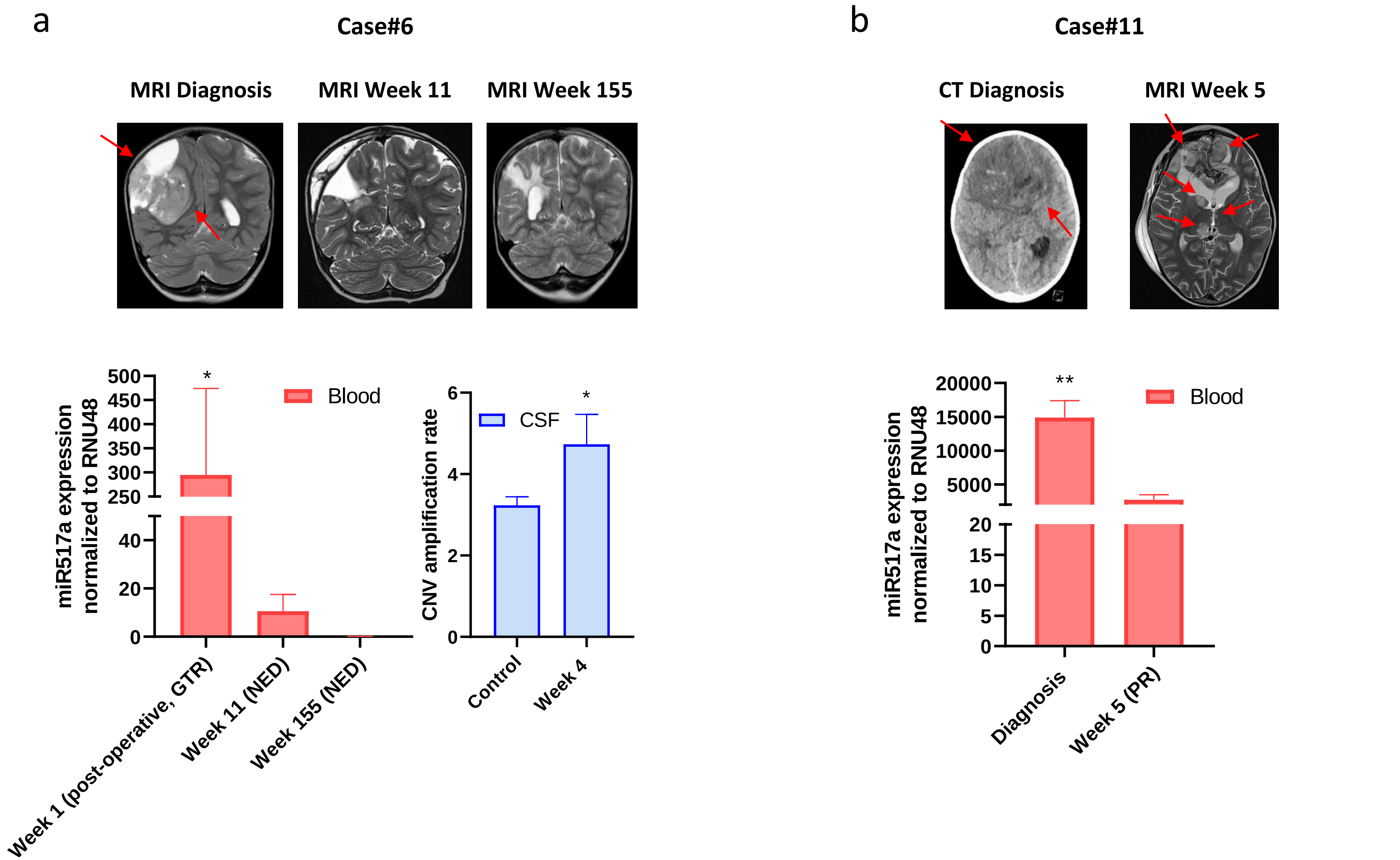

Supplementary Figure 2: Liquid biopsy detection of miRNA enables therapy monitoring in ETMR patients. ETMR cases and matched MRI images (FLAIR, upper panel) as well as longitudinal monitoring of miR517a expression in serum and CNV in CSF for a) case#6 and plasma for b) case#11.

Supplementary Figure 3

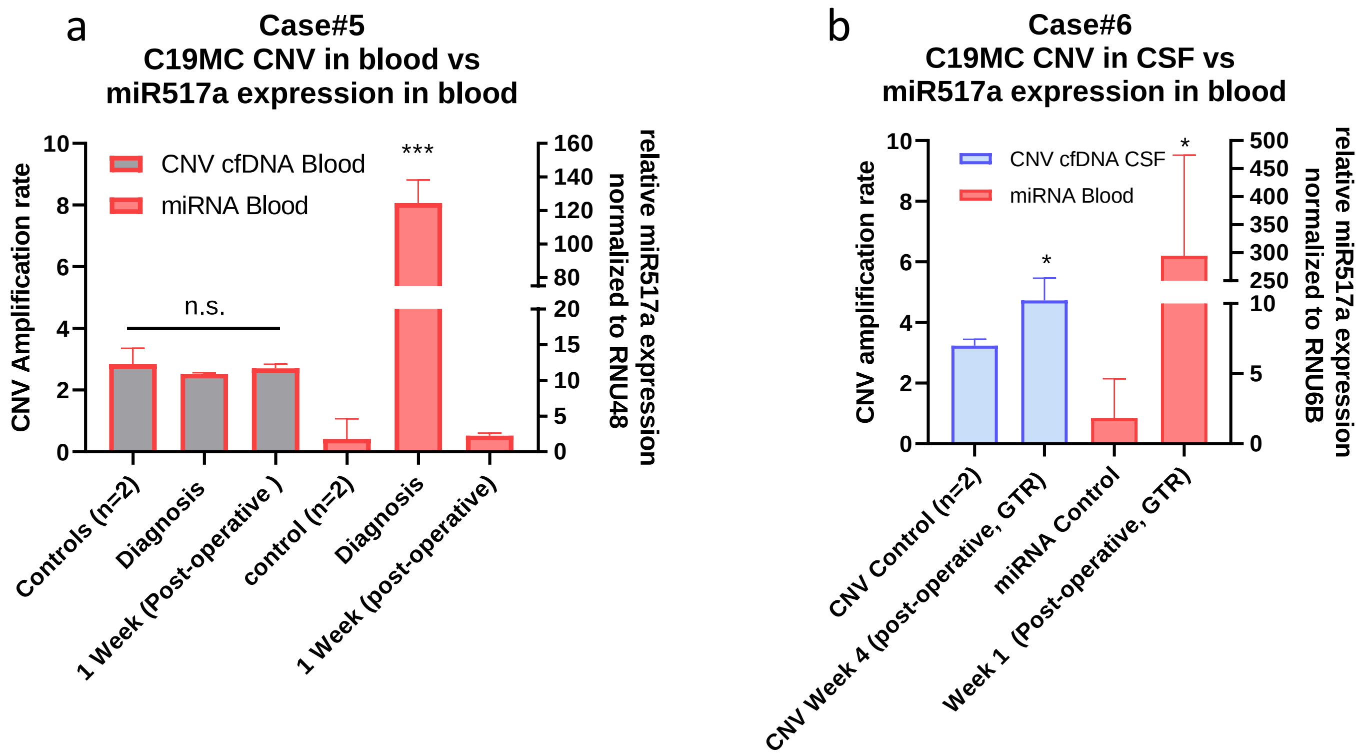

Supplementary Fig. 3: a) Detection of CNV in blood and CSF. Amplification cut off was set to 2.5. a) Case#5 CNV vs miRNA expression in blood. b) case#6 CNV of C19MC cluster in CSF vs miR517a expression in blood.

Supplementary Table 1

Detailed information on isolated blood source of patient

Case	Blood source	Isolated volume	Isolated RNA source	Sample quantity
Case#5	Serum	200µl	Small RNAs	2
Case#6	Serum	200µl	Small RNAs	3
Case#7	Plasma	200µl	Small RNAs	4
Case#9	Plasma	200µl	Small RNAs	3
Case#11	Plasma	200µl	Small RNAs	2

Supplementary Table 2

Specificity and Sensitivity of miR-517a in different analytes compard to non-tumor controls

	Liquid biomarker				
Source	Plasma	Serum	Serum	CSF	CSF
Structure	miRNA	miRNA	cfDNA	miRNA	cfDNA
Marker	miR517a	miR517a	miR517a	miR517a	miR517a
Case	5	2	1	6	6
Controls	20	20	2	3	5
GEX (fold change)	2.5 -17.000	4 - 500	n.a.	0.7- 45.000	n.a.
CNV	n.a.	n.a.	2.5	n.a.	2.3 - 5.5
Specificity	100	100	n.a.	60	80
Sensitivity	100	100	n.a.	66.66	70
Positive predictive value (PPV)	100	100	n.a.	66.66	100
Negative predictive value (NPV)	100	100	n.a.	60	71.42

Supplementary Table 3

Summary of ETMR cases with liquid biopsy analyses adapted from Mayr et al.:

Mayr L, Gojo J, Peyrl A, Azizi AA, Stepien NM, Pletschko T, et al. *Potential Importance of Early Focal Radiotherapy Following Gross Total Resection for Long-Term Survival in Children With Embryonal Tumors With Multilayered Rosettes*. Front Oncol. 2020;10:1–10.

Case	Age (months)	Gender	Location	Surgery	M-Stage	Primary CT	Focal RT (months after Dx)	Intrathecal CT	HDCT	Status/ Follow up (months)
**5**	16	f	pineal	GTR	M0	MUV ATRT	6.5	yes	no	DOD,13
**6**	38	f	parietal	GTR	M0	PEI/TMZ	1.5	yes	no	NED,87+
**7**	27	f	bifrontal	GTR	M0	PEI/TMZ	1.5	yes	no	NED,82+
**9**	19	m	pineal	PR	M0	IP-CZD	5.5	yes	no	DOD,14
**11**	27	F	bifronal	PR	M0	talazoparib/topotecan	no	no	no	DOD,5

CT, chemotherapy; RT, radiotherapy; Dx, diagnosis; HDCT, high-dose chemotherapy; GTR, gross total resection; PR, partial resection; MUV ATRT, Medical University of Vienna – Atypical teratoid rhabdoid tumor protocol (Slavc et al., Cancer Medicine 2014); PEI, cisplatin-etoposide-ifosfamide; TMZ, temozolomide; IP-CZD, polish infant IP-CZD protocol; DOD, death of disease; NED, no evidence of disease
