# Supplementary material for: Clinical applicability of miR517a detection in liquid biopsies of ETMR patients
Source: Acta Neuropathol. 2023 Apr 5;145(6):843–6. doi: 10.1007/s00401-023-02567-z (PMC10175469; doi:10.1007/s00401-023-02567-z)
Supplement: Supplementary file 2 — Supplementary file2 (PDF 120 kb) [file 401_2023_2567_MOESM2_ESM.pdf]

Clinical applicability of miR517a detection in liquid biopsies of ETMR patients

Madlener et al.

Online Supplementary Materials and Methods

Human subjects and ethical considerations

The study was approved by the local institutional review board (IRB) of the Medical University of Vienna (EK Nr. 1244/2016). Informed consent to participate in the study was obtained from patients and/or legal representatives of patients treated at the Medical University of Vienna/General Hospital of Vienna or the Kepler University Hospital Linz.

Patient samples and controls

The study included nine tumor tissues of ETMR. For control issues we included brain tissue from epilepsy surgeries (n=3), other embryonal brain tumor types (21 MB and 7 ATRT) and high-risk brain tumors (3 HGG and 6 DMG). The liquid biopsy analyses were normalized to a serum pool of 20 non-tumor bearing pediatric patients and plasma (n=3) as well as CSF samples (n=3) of medulloblastoma patients.

Tumor RNA isolation

Prior to the isolation with the RNeasy Midi Kit from Qiagen, the tumor tissue was homogenized with a precellys homogenizer from Peqlab. The total RNA isolation was performed according to the instrcutors manual and the concentration and quality was measured with a Nanodrop 1000.

NanoString nCounter miRNA Assay

100ng isolated RNA from tumor tissues was used for the NanoString Assay. We processed the assay according to the insturctors manual and run the samples with the nCounter PRO Analysis System from NanoSting and performed the analysis with the nSolver 4.0 software from NanoString.

RNA purification from liquids

Here, the Plasma/Serum RNA purification Kit from Norgene Biotek was used. A total volume of 100µl serum/plasma and 300µl of CSF were isolated for each patient and time point. The protocol was performed as described by the company with some minor modifications. Details are available upon request from the authors.

Reverse transcription

We performed the cDNA synthesis with the miRNA reverse transcription assay from Thermo Scientific with some minor modifications. We used RNU6B for tissue control and RNU48 for the liquids. Details are available upon request from the authors.

gDNA isolation from dried blood spots and digital droplet PCR

We isolated DNA from 4 punched out circles of a dried blood spot card from each patient and performed the genomic DNA isolation with the QIAamp DNA blood kit from Qiagen. ddPCR was done with predesigned and validated probes from BioRad. We purchased the miR517-FAM probe (Assay ID dHsaCNS584499451) and MDM4-HEX probe (Assay ID dHsaCP2506520) from Bio-Rad Laboratories Inc., Hercules, CA, USA) for the analysis of the copy number variations in the blood spots. The copy number variation was estimated using the following formula:

$$\text{Amplification rate} = \left(\frac{\text{Copies gene of interest}}{\text{Copies of reference gene}}\right) \text{x2}$$

Imaging data analysis

Magnetic resonance imaging obtained during routine treatment of patients was assessed by an experiences neuroradiologist (J.F-S.). Tumor volume was assessed in 3D on the basis of the Response Assessment in Pediatric Neuro-Oncology (RAPNO) criteria.
